# Neurectomy of the Inferior Branch of the Fifth Nerve

**Published:** 1889-06

**Authors:** J. H. Glass

**Affiliations:** Utica, N. Y.


ARTICLE II.

### NEURECTOMY OF THE INFERIOR BRANCH OF THE FIFTH NERVE.

BY J. H. GLASS, M. D., UTICA, N. Y.

[Read at New York State Medical Society, Albany, N. Y.]

In an examination of the literature on the subject at my command, I find no mention of the operation of excision of the inferior branch of the fifth nerve, excepting that which contemplates the opening of the inferior dental canal through the ramus of the jaw externally. This operation necessitates the division of some or all fibres of the masseter muscle, the repair of which may not always be rapid and complete owing to its almost constant and semi-involuntary action. If absolute rest is enjoined—an essential to prompt and perfect repair—much discomfort must result from masticatory disability. As to cosmetic effect, the operation is not a promising one, as a more or less ugly superficial cicatrix is inevitable, and the facial contour is frequently disturbed, following imperfect union of the muscular fibres or contractions incident to occasional pus accumulations. In consideration of these objections I desire to suggest an operation which I believe is not open to these criticisms or to others of equal importance, although my experience with the operation on the living subject is limited to the single case which is described. Anatomically I have in several instances demonstrated the practicability of the operation on the cadaver and can see no valid reason why its employment—as a substitute for the operation usually described by our text-books—should not become general.

Two years since, Theo. Walter, of Oriskany Falls, at the instance of his family physician, consulted me for relief from a persistent neuralgia of the inferior branch of the fifth nerve, of several years duration. The patient, a German of about 55 years of age, was apparently a resolute and courageous individual of naturally strong physique, but at this time was wasted and much depressed from his prolonged and almost uninterrupted pain, signified his willingness to submit to any operation however severe, and abide any result of whatever nature, provided he could obtain relief of his intolerable suffering. He declared with an earnestness that impressed one with his sincerity of purpose, that unless we did succeed his only alternative was speedy self-destruction.

The patient declining anything but a local anæsthetic, although admonished of the painful character of the nerve section, a few minims of a 4 per cent. solution of cocaine were deeply injected in the region of the dental foramen. The jaws were widely separated and held in position by an ordinary dental post placed on the side opposite to that of the proposed operation, an assistant steadying the head and controliing the tongue when necessary with a depressor, with the index finger over spine of the inferior dental foramen—a land-mark of the greatest importance in this procedure—an opening was made with angular scissors, cutting under the finger five-eighths of an inch in length backward and upward, separating the soft parts down to and about the nerve which lies in front of the artery, at this point hugging the greatest concavity of the notch; just above the spine a Theobald's strabismus hook was passed under the nerve which, isolated from the artery, was seized with Wecker's clamp strabismus hook, the nerve firmly held, a hook bistoury was passed, the back of the instrument pushing the soft parts up as high as possible; the cutting edge was turned and the upper section made by a to-and fro motion of the knife; with the trunk of the nerve still held in the clamp, it was drawn up as much as possible, and the lower section made in the same manner, completing the neucrectomy—substantially, the steps were the same in the experimental operations on the cadaver.

In the case detailed five-sixteenths of an inch of the nerve trunk was removed, which seems to have been ample, as he has had no recurrence of his neuralgic symptoms up to the present time. It should be remembered that all nervous regeneration takes place from the central end of the nerve, and that the relief promised is in constant relation to the more nearly approximate central section. The danger of wounding important vessels must not be ignored, yet I believe little risk is entailed if we bear in mind the relation of the internal maxillary artery which, near the point involved, skirts the inferior margin of the internal pterygoid muscle.—*Ohio Journal of Dental Science.*

